# New *PTEN* mutation identified in a patient with rare bilateral choroidal ganglioneuroma

**DOI:** 10.1186/s12886-020-01760-y

**Published:** 2020-12-11

**Authors:** Zhaoxin Jiang, Ting Zhang, Chonglin Chen, Limei Sun, Songshan Li, Xiaoyan Ding

**Affiliations:** State Key Laboratory of Ophthalmology, Zhongshan Ophthalmic Center, Sun Yat-sen University, Guangzhou, 510060 China

**Keywords:** Choroidal ganglioneuroma, *PTEN* mutation, Whole exome sequencing

## Abstract

**Background:**

Choroidal ganglioneuroma is an extremely rare tumor, and there is little knowledge regarding its pathogenesis. We aimed to investigate the phenotypic and genetic alterations in one sporadic patient with a rare case of bilateral choroidal ganglioneuroma.

**Methods:**

A 6-year-old boy with histological diagnosis of bilateral ganglioneuroma was recruited for the study. Comprehensive ophthalmic examinations were performed. Genomic DNA was extracted from the peripheral blood samples collected from the patient, his unaffected family members, and 200 unrelated control subjects from the same population. Whole exome sequencing was performed and raw reads were aligned to the human genome reference (hg19) using Burrows-Wheeler Aligner. DNA from all available family members was Sanger sequenced for segregation analysis.

**Results:**

Extensive bilateral retinal detachments were observed via optical coherence tomography. Diffuse thickening of choroid was identified with ultrasound B scan and magnetic resonance imaging. Genetic analysis revealed the presence of a novel heterozygous *PTEN* frameshift mutation, c.498delA (p.Thr167LeufsTer16), in exon 6. It was present in the affected individual, but not in any of the family members. Genetic analysis revealed that there was no mutation in neurofibromatosis-related genes in the family. Upon performing comprehensive systemic examinations, no obvious abnormalities in other organs were observed.

**Conclusions:**

A novel de novo *PTEN* mutation was identified in a patient with bilateral choroidal ganglioneuroma. Although *PTEN* mutations are known to induce multiple abnormalities, choroidal ganglioneuroma can be the first manifestation without abnormalities in other organs. Further studies are needed to confirm the association between choroidal ganglioneuroma and *PTEN* mutation.

## Background

Choroidal ganglioneuroma is an extremely rare disease, with only 13 reported cases [1–13]. Ganglioneuroma is a benign neurogenic tumor with an incidence of approximately 1 case per million children in the United States [14, 15]. It arises from the neural crest [16], and is considered as a subset of neuroblastomas that histopathologically consist of mature ganglion-like cells with scarce immature cells [17, 18]. The underlying mechanism of the development of ganglioneuroma is still unclear.

According to the previous reports, blindness and painful eyes were common features, and 12 out of 13 cases had neurofibromatosis type 1 (NF-1), which suggests that choroidal ganglioneuromas could be a rare manifestation within the clinicopathologic spectrum of NF-1 syndrome [19]. Recently, we encountered the first case of bilateral choroidal ganglioneuromas, and reported the multimodal imaging features in the early stage [20].

With the aim to unravel the pathogenesis of this rare disease, whole exome sequencing was carried out, and a novel phosphatase and tensin homolog (*PTEN*) mutation was identified in the patient. The results suggest that there may be an association between choroidal ganglioneuroma and the *PTEN* mutation.

## Methods

### Study participants

This study was performed as per the guidelines approved by the Ethics Committee of Zhongshan Ophthalmic Center (ZOC), Sun Yat-sen University and in accordance with the Declaration of Helsinki. Written informed consent was obtained from all subjects.

A 6-year-old boy complaining of gradual bilateral vision loss for the last 2 years presented in Pediatric Ophthalmology Department, ZOC. Comprehensive ophthalmic examinations were performed, including visual acuity, intraocular pressure (IOP), slit lamp microscopy, fundus photography (Heidelberg Engineering, Inc., Heidelberg, Germany), optical coherence tomography (OCT; Carl Zeiss Meditec, Inc., Dublin, CA, USA), ultrasound B scan (Aitomu Machinery Co. Ltd., Shanghai, China), and magnetic resonance imaging (MRI; General Electric, Milwaukee, WI). As choroidal ganglioneuroma was diagnosed based on previous choroidal biopsy, extensive examinations were performed to screen any systemic involvement. Whole exome sequencing was performed to unravel the genetic pathogenesis of this rare disease.

### DNA sample collection

**Fig. 1:**
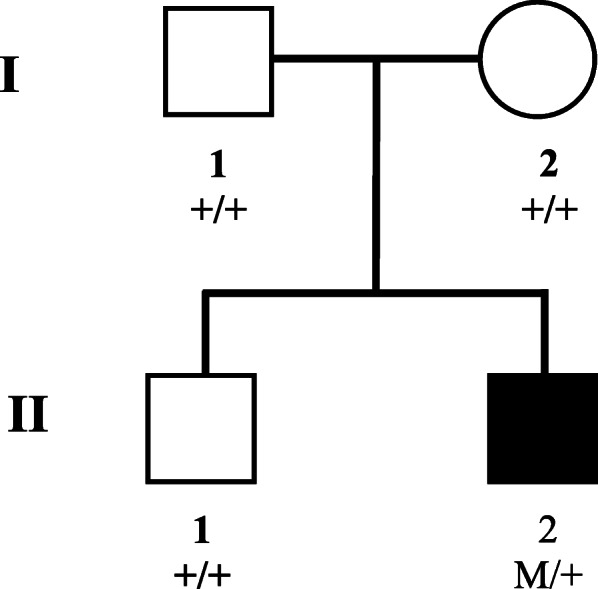
Pedigree of the family of the patient diagnosed with choroidal ganglioneuroma. Squares represent males, circles represent females, and black symbol identifies the clinically affected individual

The proband was the second child of healthy parents (Fig. 1). The parents had no knowledge of any cancer, or diseases associated with NF and PTEN hamartoma tumor syndrome. Blood samples were collected from the proband and his unaffected family members. A total of 200 individuals from the same population, who exhibited no diagnostic features of tumor, neurofibromatosis or PTEN hamartoma tumor syndrome, were recruited to serve as normal control population (41.99 ± 15.62 years old, 105 males/95 females). Genomic DNA was extracted using the TIANamp Blood DNA Kit (Tiangen Biotech, Beijing, China) according to the manufacturer’s instructions. The quantity and quality of DNA were verified with NanoDrop (2000c Model, Thermo Fisher, US).

### Library preparation and targeted sequencing

Illumina paired-end libraries were prepared using the Kapa LTP library prep kit (Roche, Basel, Switzerland) according to the manufacturer’s protocol. Briefly, genomic DNA was sheared into fragments approximately 300–500 bp in length. The DNA fragments were end-repaired and an extra ‘adenine’ base was added to the 3′ end. Illumina adapters were ligated to the ends of the DNA fragments and subsequently four cycles of PCR amplification were performed on each sample. The DNA libraries were quantified using Qubit 3.0. Pre-capture libraries were pooled together for each capture reaction. Agilent SSELXT Human All Exon V6 was used for whole exome sequencing (Agilent, Santa Clara, CA, USA). The enriched DNA library was sequenced on Illumina Xten Analyzers, at 150 cycles per read, to generate paired-end reads following the manufacturer’s standard sequencing protocols.

### Bioinformatic analysis of sequencing results

Raw reads were aligned to the human genome reference (hg19) using the Burrows-Wheeler Aligner. Single-nucleotide variants (SNVs) and insertions and deletions (InDels) were called by GATK4.0 HC. The frequency of all SNVs and InDels was annotated using the ExAC, gnomAD, HGVD, CHARGE, 1000 Genome, UK10K databases, and the internal database of Clinbytes Inc. to filter the common variants, with an allele frequency cutoff of 0.5 and 0.1% for recessive and dominant variants, respectively.

### Genetic validation

After the confirmation of pathological variants in the proband, samples from all available and consenting family members were Sanger sequenced for segregation analysis. PCR primer sets were designed via Primer3 and products were sequenced on an ABI 3700XL Genetic Analyzer. The sequences of forward and reverse primers used for amplification of *PTEN* are 5′-GGCTACGACCCAGTTACCATAG-3′ and 5′-TGGGACAGGTTCTTCCATCATC-3′, respectively.

## Results

### Ocular findings

In an initial examination of the proband 2 years ago, the best corrected visual acuity was 20/50 in the right eye and 20/32 in the left. In a more recent visit, it was hand motion/10 cm and 20/200, respectively. The IOP had fluctuated around the normal range in the past, but 40 mmHg IOP was observed in recent visit, and was controlled by administering brinzolamide-timolol eyedrops.

**Fig. 2:**
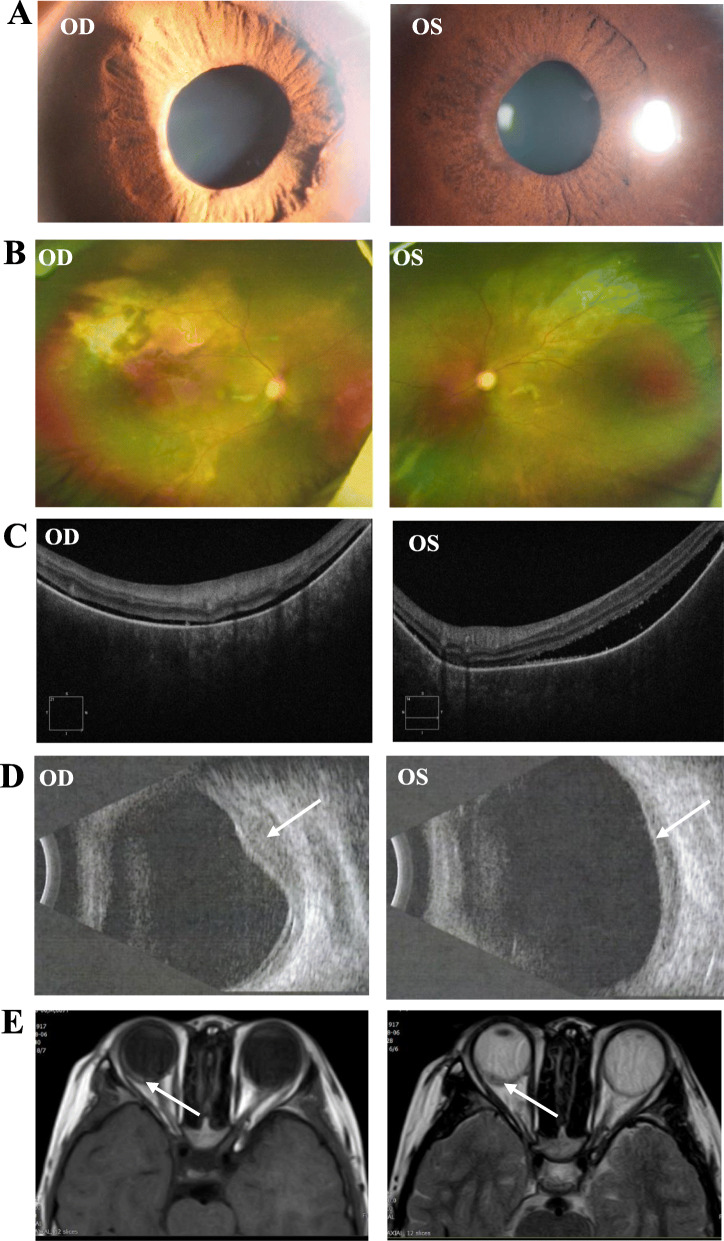
Images of comprehensive ophthalmic examinations. **a** The bilateral pupils were irregular and unable to dilate. **b** Funduscopy showed vasculitis and exudation bilaterally. **c** Bilateral retinal detachment was observed in optical coherence tomography. Choroidal vasculature was dramatically absent. **d** A dome-shaped mass bulging from choroid to retina was observed in B scan. **e** Diffuse thickening of bilateral choroids was verified with magnetic resonance imaging

Bilateral and extensive retinal detachments were observed in OCT. Diffuse thickening of choroid was identified via B scan and MRI (Fig. 2). There was a high probability of occurrence of a choroidal tumor, and a choroid biopsy confirmed the histological diagnosis of choroidal ganglioneuroma. More details on the multimodal images can be found in our previous report [20].

### Mutation screening

**Fig. 3:**
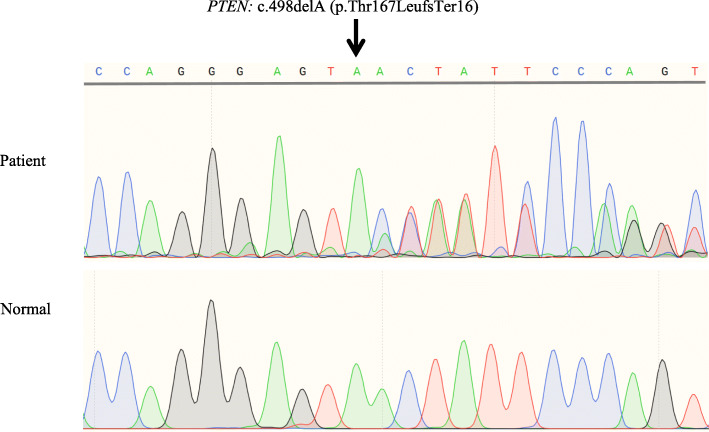
DNA sequence of a region of the *PTEN* gene in the patient and unaffected individuals. A heterozygous *PTEN* frameshift mutation c.498delA (p.Thr167LeufsTer16) in exon 6 (NM_000314) was identified in the affected individual, but not in the unaffected family members or unrelated control subjects

Genetic analysis documented the presence of a heterozygous *PTEN* frameshift mutation, c.498delA (p.Thr167LeufsTer16), in exon 6 (NM_000314) in the affected individual, but not in any of the unaffected family members. Therefore, this was considered as a de novo mutation. The identified mutation has not been reported previously and was not observed in any of the 200 unrelated control individuals from the same population (Fig. 3). The c.498delA variant causes a frameshift mutation starting with codon threonine-167, changing this amino acid to a leucine residue, and creates a premature stop codon at position 16 of the new reading frame, denoted as p.Thr167LeufsTer16. It is predicted that this variant can cause loss of normal protein function either through protein truncation or nonsense-mediated mRNA decay.

As most reported cases of choroidal ganglioneuroma showed a medical history of NF-1, genetic changes in neurofibromatosis-related genes were evaluated for all subjects. Genetic analysis showed that there was no mutation in NF1- or NF2-related genes in the proband and his unaffected family members. There was no pathogenic or a potential pathogenic mutation in NF-related genes in any of the 200 unrelated controls.

### Systemic workup

Since the patient had choroidal ganglioneuroma and the mutation in *PTEN*, it was crucial to rule out the presence of other ganglioneuromas, or PTEN related abnormalities in other tissues. The patient was subjected to comprehensive systemic examinations, including general physical examination, ultrasound scan of the thyroid gland, liver and kidney, CT scan of the mediastinum and retroperitoneum, and no obvious abnormalities were detected.

## Discussion

According to the reported studies (Table 1), most cases of choroidal ganglioneuroma shared some common features. First, most cases of uveal ganglioneuroma co-occurred with NF-1, leading to the diagnosis as NF-1with orbit-facial involvement [19]. Second, ganglioneuroma led to unilateral blindness and pain in the eye and ended up with evisceration/enucleation in all cases. Third, ganglioneuromas were diagnosed unexpectedly only after subsequent histopathological examination. Genetic examination performed in one of the cases diagnosed as Cowden syndrome revealed a mutation in *PTEN* gene.

**Table 1:** Clinical profiles of choroidal ganglioneuroma cases in literature

	Case	Sex	Age (Years)	Eye	Clinical presentation	NF-1	Treatment
1	Wolter	Female	42	Left	Cataract, glaucoma, buphthalmos	Yes	Enucleation
2	Woog et al	Female	21	Left	Cataract, retinal detachment, buphthalmos	Yes	Enucleation
3	Browstein et al	Female	< 1	Left	Congenital glaucoma, buphthalmos, plexiformneurofibroma	Yes	Enucleation
4	Shome et al	Male	11	Left	Glaucoma, buphthalmos, plexiform neurofibroma,sphenoid wing dysplasia	Yes	Evisceration
5	Ishijima et al	Male	7	Left	Cataract, retinal detachment, buphthalmos	Yes	Enucleation
6	Lad et al	Female	< 1	Right	Cataract, buphthalmos, sphenoid wing dysplasia	Yes	Enucleation
7	Yazici et al	Female	17	Left	Cataract, glaucoma, buphthalmos plexiformneurofibroma	Yes	Evisceration
8	Goyal et al	Female	< 1	Right	Buphthamos, hypoglobus, plexiform neurofibroma	Yes	Exenteration viacraniotomy access.
9	Chang et al	Male	42	Right	Cataract, optic nerve glioma, neurofibroma,	Yes	Enucleation
10	Mbagwu et al	Female	5	Right	Proptosis, glaucoma, sphenoid wing dysplasia	Yes	Enucleation
11	Abdulkader et al	Male	50	Right	Cataract, glaucoma, buphthalmos, plexiformneurofibroma, frontoethmoidal encephalocele	Yes	Orbital exenteration
12	DeParis et al	Female	5	Right	Glaucoma, retinal detachment, macrocephaly,developmental delay	No	Enucleation
13	Gilani et al	Male	7	Not mentioned	Invading plexiform neurofibroma, choroidallayer expanding	Yes	Enucleation
14	Present case	Male	6	Bilateral	Retinal detachment and diffuse choroidalthickening	No	IOP lowering medicines

In this case, retinal detachment was a major early manifestation, and choroidal tumor was suspected when the thickness of the choroid increased. There were no clinical features that supported NF-1 throughout extensive examinations. Choroidal biopsy was performed and the diagnosis of ganglioneuroma was confirmed [20]. Interestingly, this is the first case of bilateral choroidal ganglioneuroma. Thus, whole exome sequencing was performed and a de novo PTEN mutation was identified. *PTEN* is a tumor suppressor gene that classically dampens the PI3K/AKT/mTOR growth-promoting signaling cascade [21]. Loss of *PTEN* function results in increased cell proliferation, survival, and tumorigenesis [22, 23], manifesting as diverse human pathologies, and leading to the use of the umbrella term, PTEN hamartoma tumor syndrome (PHTS) [24, 25]. Thus, based on histologic features, this case was diagnosed as choroidal ganglioneuroma. Besides, based on genetic findings, this case was diagnosed as PHTS manifesting with choroidal ganglioneuroma.

The association among *PTEN*, GN, and NF is not clearly known yet, but studies have shown some common underlying pathogenic mechanisms. NF-1 is considered to be a syndrome that increases disposition to tumors, since individuals with NF-1 are 10 to 50 times more prone to a diverse spectrum of benign and malignant tumors than the general population [26, 27]. On the other hand, several reports support that there are alterations of *PTEN-*controlled signaling pathways in NF [28]. For instance, NF associated high-grade glioma models have been established by coupling complete inactivation of *NF-1* and *PTEN* genes [29, 30]. Also, several studies have demonstrated a role of neurofibromin, the protein product of *NF-1*, in controlling mammalian target of rapamycin (mTOR) signaling, which indicates the involvement of *PTEN-*related PI3K/AKT/mTOR pathway in the etiology of NF-1 [31, 32].

In the current case, it is interesting that there are no features of NF or PHTS. We speculate that this can be due to no pathogenic mutation in NF-related genes and the young age of the patient. Different cancer risks have been reported in PHTS and age is an important variable. For instance, Tan et al. studied 368 individuals with *PTEN* mutation and showed that risks of developing colorectal and kidney cancers began around the age of 40, with a lifetime risk of 9 and 34% respectively [33]. The penetrance of breast cancer is particularly elevated, beginning around the age of 30 and rising to an estimated 85% lifetime risk in females [33]. Nieuwenhuis et al. analyzed the data from 180 patients with *PTEN* mutations and reported that cumulative risk of developing any cancer was 9% at the age of 30, but it increased to 55.7% at the age of 60 in males [34]. Thus, genetic study is important for the diagnosis, as *PTEN* mutations are known to increase risks for multiple common cancers. Lifetime cancer risk estimates and close follow-ups are highly recommended for the proband in the present case.

One limitation of the present study is the limited case number. Choroidal ganglioneuroma is an extremely rare disease and most reported cases are not diagnosed until evisceration/enucleation. Therefore, it might be very difficult to recruit enough cases for a case-series study. Furthermore, biopsy and genetic testing are critical for the early and accurate diagnosis of choroidal ganglioneuroma.

## Conclusion

A novel de novo *PTEN* mutation was identified in a bilateral choroidal ganglioneuroma patient. Although *PTEN* mutation has been considered to induce multiple abnormalities, choroidal ganglioneuroma can be the first clinic manifestation without abnormalities in other organs. Further studies are needed to confirm this new association between choroidal ganglioneuroma and the *PTEN* mutation.

## Data Availability

All the data used to support the findings of this study are included within the article and are available from corresponding author by a reasonable request.
